# Supplementary material for: Student experiences with a molecular biotechnology course containing an interactive 3D immersive simulation and its impact on motivational beliefs
Source: PLoS One. 2024 Jul 25;19(7):e0306224. doi: 10.1371/journal.pone.0306224 (PMC11271915; doi:10.1371/journal.pone.0306224)
Supplement: S1 Table — (DOCX) [file pone.0306224.s001.docx]

**S1** **Table.** Cronbach’s alpha (α) measure of scale reliability for biotechnology self-efficacy and science identity.

|  | Survey | | |
| --- | --- | --- | --- |
| Measure | Pre-Lab | Post-Lab | End of semester |
| Biotechnology Self-Efficacy | .84 | .90 | .89 |
| Science Identity | .88 | - | .94 |
