# Supplementary material for: Student experiences with a molecular biotechnology course containing an interactive 3D immersive simulation and its impact on motivational beliefs
Source: PLoS One. 2024 Jul 25;19(7):e0306224. doi: 10.1371/journal.pone.0306224 (PMC11271915; doi:10.1371/journal.pone.0306224)
Supplement: S2 Table — (DOCX) [file pone.0306224.s002.docx]

**S2** **Table.** Comparisons of perceptions towards the virtual interactive cell culture simulation post lab and end of semester.

| **Item** | **Post-Lab** | **End of Semester** | **Repeated-measures MANCOVA** |
| --- | --- | --- | --- |
| I was more engaged in learning as a result of using the virtual interactive cell culture simulation. | 3.08 (1.21) | 3.08 (1.10 ) | F(1,128) = .08, p = .78 |
| The virtual interactive cell culture simulation required me to think critically. | 3.45 (1.25) | 3.33 (1.21 ) | F(1,128) = 2.39, p = .12 |
| The virtual interactive cell culture simulation helped me make connections between my prior knowledge and new knowledge. | 3.61 (1.11) | 3.52 (1.10) | F(1,128) = 2.75, p = .10 |
| The virtual interactive cell culture simulation helped me better understand the importance of sterile mammalian cell culture technique | 3.52 (1.22) | 3.45 (1.21) | F(1,128) = .80, p = .37 |
| Virtual interactive cell culture simulation has clear connections to real-world applications | 3.75 (1.08) | 3.80 (1.04) | F(1,128) = .21, p = .65 |
| *Note:* post-lab and end of semester columns include mean and (standard deviation) | | | |
