# Supplementary material for: Student experiences with a molecular biotechnology course containing an interactive 3D immersive simulation and its impact on motivational beliefs
Source: PLoS One. 2024 Jul 25;19(7):e0306224. doi: 10.1371/journal.pone.0306224 (PMC11271915; doi:10.1371/journal.pone.0306224)
Supplement: S1 Appendix — (DOCX) [file pone.0306224.s003.docx]

# **Supporting Information**

**S1 Appendix.** Pre-lab, post-lab and end of semester survey questions.

**Self-Efficacy (Baldwin et al. (1999)) (pre-lab, post-lab, and end of semester)**

The following statements pertain to your confidence level in the following **biotechnology skills and abilities**. For each question, think about your level of mastery in carrying out a given task. There are no right or wrong answers. (Scale- Strongly disagree to Strongly Agree)

To what extent are you confident that you could/will:

- read the procedures for an experiment and feel sure about conducting the experiment on your own.
- write a scientific laboratory report.
- be successful in another biotechnology course.
- analyze a set of data (i.e., look at the relationships between variables).
- ask a meaningful question that could be answered experimentally using biotechnology techniques.
- be successful in this biotechnology course.
- troubleshoot a molecular biotechnology experiment that did not work.
- design strategies to manipulate DNA to create new molecules.

**Science Identity (Chemers et al. (2010)) (pre-lab and end of semester)**

The following statements ask you to think about yourself and **your personal identity**. We want to understand how much being a scientist is part of who you are. For each statement, indicate your level of agreement from strongly disagree to strongly agree. Remember, there are no right or wrong answers. (Scale- Strongly disagree to Strongly Agree)

- In general, being a scientist is an important part of my self-image.
- I have a strong sense of belonging to the community of scientists.
- Being a scientist is an important reflection of who I am.
- I have come to think of myself as a “scientist.”
- I feel like I belong in the field of science.
- I am a scientist.

**General Perceptions (post-lab and end of semester)**

For each statement below, rate your level of agreement based on your experience using the **virtual interactive cell culture simulation**. (Scale- Strongly disagree to Strongly Agree)

- I was more engaged in learning as a result of using the virtual interactive cell culture simulation
- The virtual interactive cell culture simulation required me to think critically
- The virtual interactive cell culture simulation helped me make connections between my prior knowledge and new knowledge
- The virtual interactive cell culture simulation helped me better understand the importance of sterile mammalian cell culture technique
- Virtual interactive cell culture simulation has clear connections to real-world applications

**Open-Ended Feedback (end of semester)**

Please provide 1-2 things that you have found most helpful about the **virtual interactive cell culture simulation**. (Open-Ended)

Please provide 1-2 ways you would like to improve the **virtual interactive cell culture simulation and related activities** to help you learn better. (Open-Ended)
